# Locus Coeruleus Degeneration in Essential Tremor With Mild Cognitive Impairment: A Neuromelanin MRI Study

**DOI:** 10.1111/cns.70214

**Published:** 2025-01-08

**Authors:** Yuelin Fang, Cheng Zhou, Bingting Zhu, Jiasi Liu, Sicheng Liu, Xiaojun Guan, Tao Guo, Xiaojun Xu, Minming Zhang, Jun Tian, Xinzhen Yin, Baorong Zhang, Guohua Zhao, Yaping Yan

**Affiliations:** ^1^ Department of Neurology, the Fourth Affiliated Hospital, International Institutes of Medicine Zhejiang University School of Medicine Hangzhou China; ^2^ Department of Radiology, the Second Affiliated Hospital Zhejiang University School of Medicine Hangzhou China; ^3^ Department of Neurology, the Second Affiliated Hospital Zhejiang University School of Medicine Hangzhou China

**Keywords:** essential tremor, locus coeruleus, mild cognitive impairment, neuromelanin‐sensitive magnetic resonance imaging

## Abstract

**Objective:**

Our aim was to research the neuromelanin‐sensitive magnetic resonance imaging (NM‐MRI) features of the locus coeruleus (LC) in essential tremor (ET) patients of various cognitive states and to explore the relationships between these features and cognition.

**Methods:**

We recruited three groups of participants, including 30 ET patients with mild cognitive impairment (ET‐MCI), 57 ET patients with normal cognition (ET‐NC), and 105 healthy controls (HCs). All participants underwent MRI scanning and clinical evaluation. Through NM‐MRI images, we compared the contrast‐to‐noise ratio of LC (CNR_LC_) between groups and evaluated the relationships between CNR_LC_ and cognitive scales.

**Results:**

Compared to HCs, ET‐MCI patients had a substantially lower CNR_LC_ value (*p* = 0.017). The CNR_LC_ of ET‐NC patients was intermediate between that of ET‐MCI patients and HCs. Furthermore, a partial correlation analysis in ET‐MCI patients, controlling for age, gender, and education level, showed that higher CNR_LC_ values correlate with better performance on the Montreal cognitive assessment test and the trail making test A.

**Conclusion:**

LC degeneration in ET patients may partially contribute to cognitive decline, suggesting that the LC norepinephrine system deserves further research on the mechanism of cognitive decline of ET patients as well as the development of targeted drugs.

## Graphical abstract

Using neuromelanin‐sensitive‐MRI (NM‐MRI) analysis, we found the integrity of locus coeruleus (LC) was decreased in ET patients and be more pronounced in ET‐MCI patients. The decreased integrity of LC was correlated with the severity of cognitive functions, suggesting some pathological changes of LC contribute to the cognitive impairment in ET.

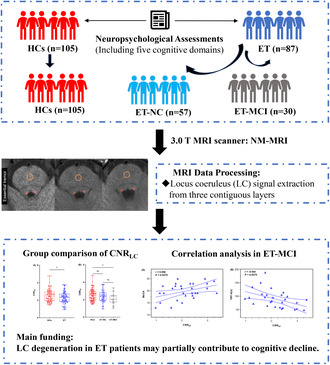

## 1 Introduction

With a global prevalence of about 0.9% among the general population, essential tremor (ET) is a highly prevailing movement disorder [1]. Its hallmark feature is kinetic tremor involving the hands and arms. Also, the tremor can eventually spread to the head, jaw, voice, and other regions [2]. It is worth noting that the definition of ET is crucial, as it has significant clinical implications. With a mass of evidence suggesting that ET is a heterogeneous disease [3, 4, 5, 6], the 2018 Movement Disorder Society Consensus Statement proposed a new concept called essential tremor‐plus (ET‐plus), which is distinct from pure ET and is characterized by the existence of additional neurological traces of unknown relevance (e.g., mild cognitive impairment, impaired tandem gait, or other signs of uncertain significance) [7].

The fact that the definition of this novel idea is based on clinical characteristics rather than etiology has also generated debate [8]. Because non‐motor symptoms were present in addition to tremors in earlier studies [9, 10, 11], partial ET patients would need to have their condition reclassified as ET‐plus. Owing to the clinical heterogeneity, it is not surprising that pathological studies were not consistent among ET patients [12]. Some studies suggest that there are variances in the cerebellar pathology observed in ET and ET‐plus, while others have not found any pathological differences [13]. Further clarification is needed for further research.

A national survey in Mainland China found that among the survey population, mild cognitive impairment (MCI) (48.2%) is the most usual neurological additional feature in ET‐plus (*n* = 614) [14]. Previous studies have shown that patients with ET have greater cognitive deficits than those observed in controls and seem to be progressive [15]. But the pathogenesis of the cognitive deficits of ET also remains unclear like the tremor. Two types of pathology are proposed for ET: A variant of reduced Purkinje cells in the cerebellum and a variant of Lewy body degeneration in the locus coeruleus (LC) [16, 17, 18]. Numerous studies have reported cerebellar injury in ET patients [19, 20, 21], patients with head tremors exhibited a more significant cerebellar atrophy pattern [22, 23], providing evidence that ET maybe a neurodegenerative disease affecting the cerebellum. In addition to motor function, the cerebellum is also linked to cognitive processes. Cerebellar cognitive affective syndrome (CCAS), which is characterized by a range of cognitive and affective symptoms, commonly seen in patients with cerebellar disorders [24]. A study used the CCAS‐Scale to assess cognitive functions in 20 patients with ET and found that 13 of these individuals had a definite CCAS [25]. It is worth considering whether cerebellar damage in ET patients may also result in cognitive decline. In addition, based on neuromelanin‐sensitive magnetic resonance imaging (NM‐MRI), a magnetic resonance sequence detects the byproduct of noradrenaline (NE) called neuromelanin in vivo and allows us to make quantitative measurements of LC [26, 27, 28]. A recent study found that the contrast‐to‐noise ratio of LC (CNR_LC_) obtained from MRI scans in ET was significantly lower than that in controls. However, no relationships between CNR_LC_ and the scale reflecting tremor severity were observed [29], indicating some unknown significance of LC underline ET.

The LC is the brain's main source of NE and regulates a wide range of cognitive functions [30]. Previous researches revealed that the reduction of NE input to the basal forebrain might contribute to cognitive deterioration [31, 32]. Apart from the fact that the number of LC neurons declines with age [33, 34], pathological changes in the LC have been reported in the early stage of neurodegenerative diseases such as Alzheimer's disease (AD) [35] and Parkinson's disease (PD) [36], leading to the point that the LC may be a susceptible target for pathology. Multimodal magnetic resonance imaging showed a link between LC and cognition among these diseases [37, 38]. Considering the significant damage to the LC in ET patients [29], we speculate that the LC may be involved in the cognitive impairment of ET patients.

Through this study, we aimed to investigate the integrity of LC in ET patients with different cognitive states and explore its association with cognition. Meanwhile, we analyzed the differences in cerebellar volumes among ET patients, which may also have an impact on cognition.

## 2 Method

### 2.1 Participants

From March 2019 to April 2023, approved by the Ethics Committee of The Second Affiliated Hospital of Zhejiang University School of Medicine, we recruited 87 ET patients and 105 age, sex, and education‐matched healthy controls (HCs) in the Department of Neurology and obtained handwritten informed consent from each subject. Two neurologists made the diagnosis based on the Consensus Statement [7]. We excluded subjects with other neurological or psychiatric disorders, alcoholism, brain damage, or contraindications for MRI scanning.

### 2.2 Clinical Assessment

Every participant underwent a thorough cognitive evaluation that included tests of global cognitive function and a battery of neuropsychological assessments that addressed all five cognitive domains. Global cognitive function was evaluated using the mini‐mental state examination (MMSE) and Montreal cognitive assessment (MoCA). The details of the neuropsychological battery are as follows: (1) Memory was assessed using the auditory‐verbal learning test (AVLT) with delayed recall and a delayed recall subset of the MoCA test [39]. (2) Language was measured with the 30‐item Boston naming test (BNT) and the animal fluency test (AFT). (3) Visual–spatial function was assessed using the clock drawing test (CDT) and cube copying test of the MoCA test. (4) Attention and working memory were assessed using the trail making test part A (TMT‐A) and the symbol digit modality test (SDMT). (5) Executive function was assessed using the trail making test part B (TMT‐B) and the digit span backward. Following the Level II diagnostic criteria proposed by the 2012 Movement Disorder Society (MDS) Task Force, ET patients with mild cognitive impairment (ET‐MCI) were identified when impairment was 1.5 standard deviations (SDs) below the normative mean on at least two tests of the detailed cognitive battery [40], either within a single cognitive domain or across multiple cognitive domains. Patients with ET who didn't meet the requirements for ET‐MCI were labeled as ET with normal cognition (ET‐NC). Given that amnestic MCI (aMCI) is perhaps more likely to progress to AD, we further subdivided our ET‐MCI patients into aMCI when impairment was 1.5 SDs below the normative mean on at least one test of the memory domain [41], including the AVLT with delayed recall and a delayed recall subset of the MoCA test. Those without memory impairment are classified as non‐amnestic MCI (naMCI). The tremor research group essential tremor rating scale (TETRAS), which includes two parts: the activities of daily living scale (ADL) and the executive scale (EXEC), was used to assess the severity of tremors and their impact on daily life.

### 2.3 MRI Acquisition

All participants were scanned using a 3.0 Tesla MRI scanner with an eight‐channel head coil (Discovery MR750, GE Healthcare). Three‐dimensional T1‐weighted images were acquired using a fast spoiled gradient recall sequence with the following parameters: repetition time (TR), 7.336 ms; Echo time (TE), 3.036 ms; Inversion time, 450 ms; flip angle (FA), 11°; Field of View (FOV), 260 × 260 mm [2]; matrix, 256 × 256; slice thickness, 1.2 mm; Number of slices, 196 (sagittal); Scan time, 5 min 53 s. NM‐MRI was obtained using a T1‐weighted fast spin echo sequence: TR, 600 ms; TE, 18.6 ms; FA, 77°; FOV, 220 × 220 mm [2]; matrix, 512 × 512; slice thickness, 3 mm with no slice gap; Number of slices, 17 (axial); Scan time, 10 min 27 s. The top of the basal ganglia to the base of the medulla oblongata was the scanning coverage, and the acquisition plane was oriented orthogonal to the brainstem.

### 2.4 Imaging Assessment of NM‐MRI

**FIGURE 1:**
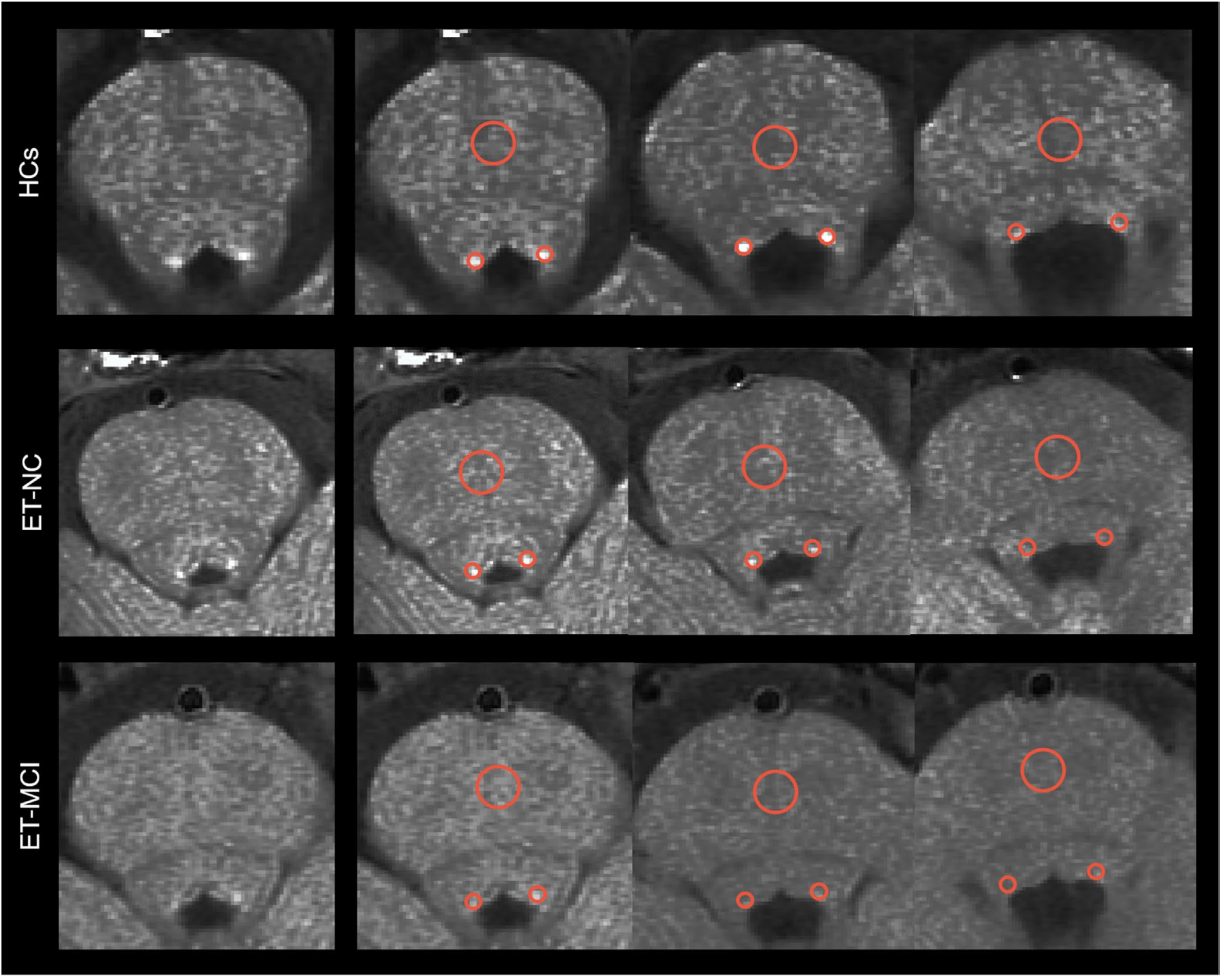
The anatomical location of the LC in HCs, ET‐NC, and ET‐MCI. The first image of each row shows the anatomical location of the LC without ROI from an individual of each group. The next three images of each row show signal intensity measurements of the LC (two small red circles) and PT (a large red circles) from the same individual for three contiguous layers. ET‐MCI, Essential tremor patients with mild cognitive impairment; ET‐NC, Essential tremor patients with normal cognition; HCs, Healthy controls; LC, Locus coeruleus; PT, Pontine tegmentum; ROI, Region of interest.

Being blinded to subjects' information, two authors (Y.L.F. and S.C.L.) performed manual measurements of LC on ITK‐SNAP (https://sourceforge.net/projects/itk‐snap/). Both of them completed the manual segmentation of the entire cohort independently. The LC was located symmetrically in the bilateral areas of the dorsal pons, adjacent to the fourth ventricle. Locations of the highest signal intensity (SI) in three contiguous layers from the level of the inferior colliculi to the superior cerebellar peduncles were identified as LC. We delineated circular regions of interest (ROIs) of bilateral LC and midparts of the pons (PT) (as contrast area) in the same consecutive slices. When the signal was considerably diminished, the ROIs were positioned at the LC's predetermined anatomical location (Figure 1). For bilateral LC, PT, the mean and standard deviation (SD) of SI were calculated. The CNR_LC_ was calculated as (SI_LC_−SI_PT_)/SD_PT_ and the averaged CNR_LC_ value from two measurements was used for further analysis. A lower CNR value indicates more severe degeneration. The measurement's intraclass correlation coefficient was 0.866.

### 2.5 Cerebellar Volumes Extraction

Using the SPM 12 toolbox (https://www.fil.ion.ucl.ac.uk/spm/software/spm12/), we completed the processing of VBM (voxel‐based morphometry) data. First, the T1 images were oriented so that the anterior commissure was placed at the origin of the 3D MNI coordinate system. Preprocessing included denoising, correcting for intensity inhomogeneity and linear intensity scaling. Then, using SPM 12 default unified segmentation, we normalized T1 images to a template space and segmented them into white matter (WM), gray matter (GM), and cerebrospinal fluid (CSF). The images were normalized to MNI space using diffeomorphic anatomical registration through exponentiated Lie algebra (DARTEL) algorithm [29, 42]. A 3D Gaussian smoothing kernel was used to smooth the normalized GM images with a full‐width at half maximum of 6 × 6 × 6 mm^3^. Finally, cerebellar volumes were extracted using the SUIT atlas.

### 2.6 Statistical Analysis

The statistical package for the social sciences (SPSS), version 26, was used to compare differences between groups. The Kolmogorov–Smirnov test was used to determine the normality of the distribution of the variables. Demographic data, clinical variables, CNR_LC_ values, and cerebellar volumes were compared using Student's *t*‐test or one‐way analysis of variance (ANOVA) for normally distributed continuous variables, and Mann–Whitney U test or Kruskal–Wallis test for continuous variables that were not normally distributed. Differences in gender distribution were compared using the chi‐square test. The two‐tailed *p* value is corrected by Bonferroni correction and statistical significance was set at *p* < 0.05. Partial correlation analysis was used to examine the correlation between CNR_LC_ and clinical variables in patients with age, gender, and education setting as covariates. For multiple comparison correction, FDR correction was performed and a *p* value < 0.05 was considered significant. Continuous variables were expressed as means ± standard deviations or medians with interquartile range according to the normal distribution.

## 3 Results

### 3.1 Demographic, Clinical, and CNR_LC_ Features

**FIGURE 2:**
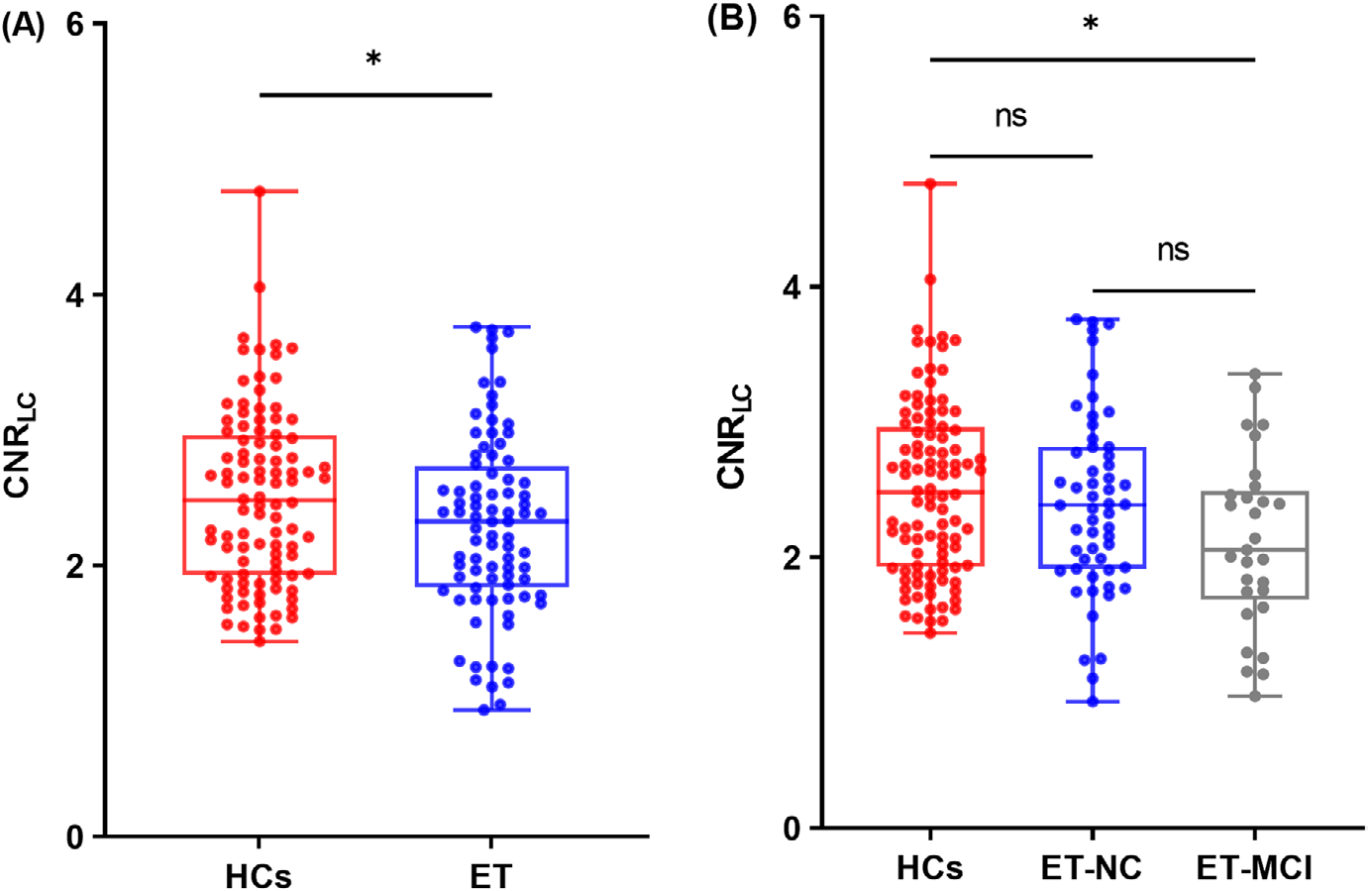
Comparisons of CNR_LC_ among groups. (A)The results of t test for CNR_LC_ between two groups. (B) The results of multiple comparisons for CNR_LC_ among three groups after covariance analysis. CNR_LC_, Contrast‐to‐noise ratio of the locus coeruleus; ET‐MCI, Essential tremor patients with mild cognitive impairment; ET‐NC, Essential tremor patients with normal cognition; HCs, Healthy controls; *p < 0.05.

From March 2019 to April 2023, 87 ET patients and 105 age, sex, and education‐matched HCs were enrolled. In terms of sex, age, and level of education, there was no statistically significant difference found between all ET patients and HCs. The CNR_LC_ of the ET group was significantly lower than that of HCs (*p* = 0.039, Figure 2A).

According to the diagnostic criteria, the participants were divided into three groups: ET‐MCI (*n* = 30), ET‐NC (*n* = 57), and HCs (*n* = 105). There was no significant difference in age, sex and level of education between groups (*p* > 0.05, Table 1). Compared to ET‐NC and HCs, the ET‐MCI group performed worse in all baseline cognitive tests. One‐way ANOVA showed that CNR_LC_ was significantly different between the three groups (*p* = 0.022, Figure 2B). Post hoc analysis showed that the CNR_LC_ in the ET‐MCI group was significantly lower than that of HCs (*p* = 0.017, Figure 2B), whereas between the ET‐MCI group and the ET‐NC group no significant differences were found (*p* = 0.193, Figure 2B) or between the ET‐NC group and HCs (*p* = 1.000, Figure 2B).

**TABLE 1:** The demographic, clinical, and CNR_LC_ characteristics of HCs, ET‐NC, and ET‐MCI.

	HCs (*n* = 105)	ET‐NC (*n* = 57)	ET‐MCI (*n* = 30)	*p*		Post hoc tests *p*	
				HC vs. ET‐NC vs. ET‐MCI	HC vs. ET‐NC	HC vs. ET‐MCI	ET‐NC vs. ET‐MCI
Age (years)	61.02 ± 7.10	59.06 ± 8.71	60.81 ± 6.62	0.277^a^			
Sex (M/F)	49/56	24/33	16/14	0.605^c^			
Education (years)	9.00 (4.00)	9.00 (3.00)	8.00 (3.50)	0.075^b^			
CNR_LC_	2.50 ± 0.65	2.39 ± 0.67	2.12 ± 0.64	0.022^a^**	1.000	0.017*	0.193
TETRAS‐ADL	—	13.07 ± 7.78	14.73 ± 10.88	0.413^a^			
TETRAS‐EXEC	—	17.03 ± 6.41	18.57 ± 9.32	0.367^a^			
MMSE	29.00 (2.00)	29.00 (2.00)	28.00 (3.00)	< 0.001^b^***	0.208	0.008**	< 0.001***
MoCA	26.00 (4.00)	26.00 (2.00)	19.00 (5.00)	< 0.001^b^***	1.000	< 0.001***	< 0.001***
**Memory function**							
Delayed recall (AVLT)	6.25 ± 2.77	5.05 ± 2.18	2.63 ± 1.75	< 0.001^a^***	0.012*	< 0.001***	< 0.001***
Delayed recall (MoCA)	3.00 (2.00)	3.00 (2.00)	1.00 (2.00)	< 0.001^b^***	1.000	< 0.001***	< 0.001***
**Language function**							
BNT	26.00 (5.00)	25.50 (4.00)	22.00 (6.00)	< 0.001^b^***	0.913	< 0.001***	< 0.001***
AFT	18.00 (7.00)	16.00 (4.00)	11.00 (7.00)	< 0.001^b^***	0.126	< 0.001***	0.001**
**Visuospatial function**							
CDT	10.00 (0.00)	10.00 (0.00)	8.00 (4.00)	< 0.001^b^***	0.919	< 0.001***	< 0.001***
Cube copy	20.00 (3.00)	20.00 (3.00)	15.00 (13.00)	0.001^b^**	1.000	0.001**	< 0.001***
**Attention and working memory function**							
TMT‐A(s)	55.00 (25.75)	54.50 (22.25)	82.00 (44.72)	< 0.001^b^***	1.000	< 0.001***	< 0.001***
SDMT	41.98 ± 10.54	39.20 ± 9.25	28.52 ± 9.89	< 0.001^a^***	0.029*	< 0.001***	< 0.001***
**Executive function**							
TMT‐B(s)	143.50 (72.75)	130.00 (70.50)	193.00 (81.50)	< 0.001^b^***	1.000	< 0.001***	< 0.001***
Digit span backward	4.00 (1.00)	4.50 (1.00)	4.00 (3.00)	0.068^b^			

*Note:* Data are expressed as means ± standard deviations or medians (interquartile range) according to the normal distribution. *p < 0.05; **p < 0.01; ***p < 0.001.

Abbreviations: AFT, Animal Fluency Test; AVLT, Auditory Verbal Learning Test; BNT, 30‐item Boston Naming Test; CDT, Clock‐Drawing Test; CNR_LC_, Contrast‐to‐noise ratio of the locus coeruleus; ET‐MCI, Essential tremor patients with mild cognitive impairment; ET‐NC, Essential tremor patients with normal cognition; HCs, Healthy controls; MMSE, Mini Mental State Examination; MoCA, Montreal Cognitive Assessment; SDMT, Symbol Digit Modality Test; TETRAS‐ADL, the Tremor Research Group Essential Tremor Rating Scale‐Activities of Daily Living scale; TETRAS‐EXEC, the Tremor Research Group Essential Tremor Rating Scale‐Executive scale; TMT‐A, Trail Making Test part A; TMT‐B, Trail Making Test part B.

^a^ Comparison performed using a parametric test (Student's *t* test or 1‐way ANOVA, as appropriate).

^b^ Comparison performed using nonparametric test (Mann–Whitney *U*‐test or Kruskal–Wallis test, as appropriate).

^c^ Comparison using chi‐square test.

We further subdivided our ET‐MCI patients into aMCI and naMCI. We found there was no significant difference in CNR_LC_ between aMCI and naMCI groups, although the naMCI group showed lower CNR_LC_ values (Table S1).

### 3.2 Correlation Analyses Between CNR_LC_ and Cognition Tests

**FIGURE 3:**
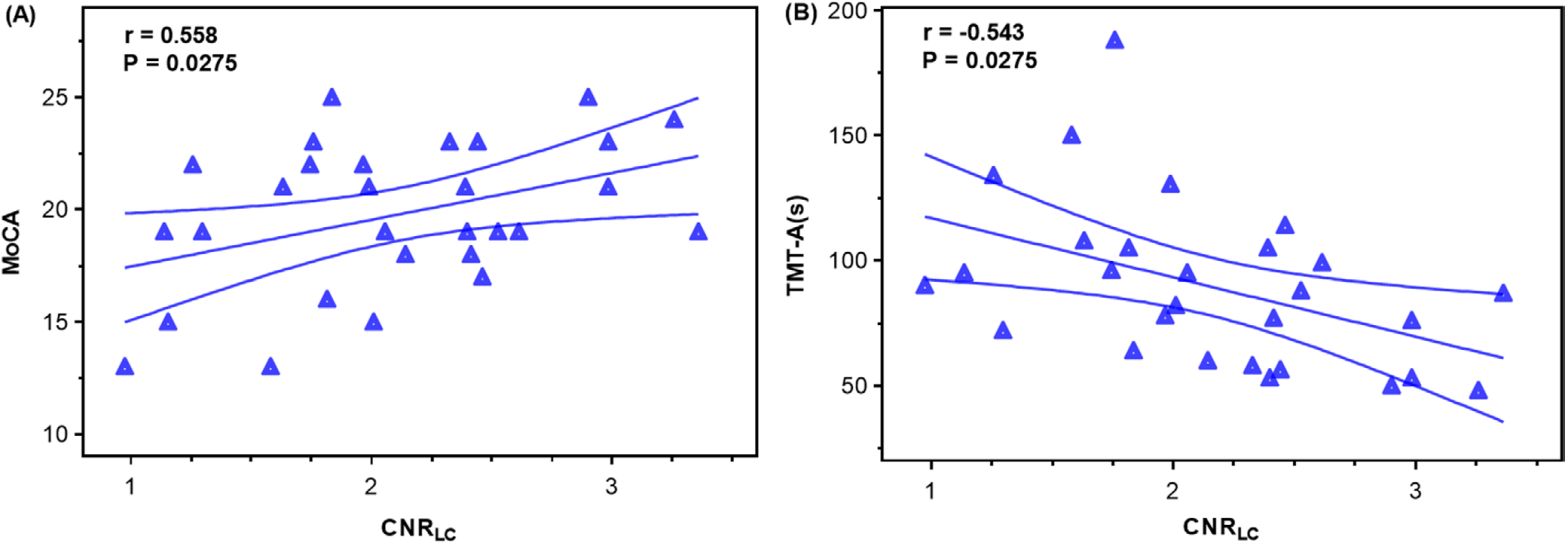
Correlation analysis between CNR_LC_ and cognitive scales in ET‐MCI patients. (A) Significantly positive correlation between CNR_LC_ and MoCA scores. (B) Significantly negative correlation between CNR_LC_ and TMT‐A time (in seconds). CNR_LC_, Contrast‐to‐noise ratio of the locus coeruleus; ET‐MCI, Essential tremor patients with mild cognitive impairment; MoCA, Montreal Cognitive Assessment; TMT‐A, Trail Making Test part A.

Correlation analysis between the CNR_LC_ and all neuropsychological examinations was performed in all ET patients. After FDR correction, the CNR_LC_ was significantly positively correlated with the MoCA scores (*r* = 0.558, *p* = 0.0275, Figure 3A) and significantly negatively correlated with the TMT‐A time (in seconds), a scale reflecting attention and working memory function (*r* = −0.543, *p* = 0.0275, Figure 3B) in ET‐MCI patients. No significant correlation was observed between the CNR_LC_ and any cognitive tests in ET‐NC patients (*p* > 0.05).

### 3.3 Cerebellar Volumes Analyses and Its Relationships With Cognition

Using the SUIT atlas, we analyzed the differences in cerebellar volumes between ET patients and HCs. We found that the volume of the left IX of cerebellum was smaller in ET patients than in HCs, and the difference was statistically significant (*p* = 0.042). However, further subgroup analysis showed no significant difference of this region between ET‐NC and ET‐MCI (*p* = 0.665). Within the ET‐MCI group, partial correlation analysis controlling for age, gender, and education level did not find a correlation between cerebellar left IX volume and cognitive scales.

### 3.4 The Relationships Between CNR_LC_ and Severity of Tremor in ET Subgroups

For ET patients, we used TETRAS score to evaluate tremor severity. We found there was no significant difference in TETRAS score between ET‐NC and ET‐MCI groups, both in TETRAS‐ADL and TETRAS‐EXEC score (*p* = 0.413, *p* = 0.367, Table 1). Besides, no significant correlation was observed between CNR_LC_ values and TETRAS score (ADL and EXEC) controlling age, education, gender as covariates of no interest in ET‐NC or ET‐MCI groups, which replicates the finding reported by Lv et al. [29].

## 4 Discussion

In this study, we examined the integrity of LC in ET patients and HCs. The integrity of LC in ET patients was significantly decreased than that of HCs. Further group comparison showed that the ET‐MCI patients had significantly decreased LC integrity compared to ET‐NC and HCs, indicating that the degeneration of LC may result in cognitive deficit in ET patients. In addition, through correlation analysis, we established the link between MoCA scores and TMT‐A time scores with CNR_LC_ of ET‐MCI patients. The MoCA test includes multiple cognitive domains that reflect the composite level of cognitive deficit, and the TMT‐A time scores reflect attention and working memory function. Further analysis did not reveal any significant differences in CNR_LC_ between aMCI and naMCI groups, although the naMCI group showed lower CNR_LC_ values. We found that the volume of the left IX of the cerebellum was smaller in ET patients than in HCs. However, further subgroup analysis showed no significant difference in this region between ET‐NC and ET‐MCI, and no associations were found between cerebellar volumes and cognitive function. Due to the small sample, these results need further study.

Located deep in the pons, LC is situated on the lateral margin of the fourth ventricle and provides the far‐reaching NE neurotransmitter system of the brain. While NE is primarily known for its function in blood pressure regulation and behavioral arousal, it also plays a crucial role in cognitive functions [30]. Previous researches showed that LC is where Alzheimer's disease pathology first appears [43, 44] and suggested that greater LC integrity was linked to better cognitive performance and slower cognitive decline [45]. Given the essential role of LC in multiple physiological activities and diseases, adequate MRI techniques to assess LC integrity in vivo is imperative for clinicians to quantify disease severity and provide insight into treatment response and prognosis. Based on the theory that NM, a pigment generated as a byproduct of catecholamine synthesis, can function as an endogenous MRI contrast agent, NM‐MRI enables the visualization of LC [46]. Because it is not well cleared out by the cell, NM accumulates over the lifespan unless the neuron dies [47]. An inverted U‐shaped curve of NM intensity throughout the lifespan was strongly proposed by previous studies [48]. And pathological and MRI studies indicated that LC NM intensity reached the peak around the age of 50–60 [48, 49]. It is noteworthy that patients with neurodegenerative illnesses have considerable reductions in LC NM signal [50, 51], suggesting that the NM‐related contrast shown in MRI images is indicative of a loss of NM‐containing neurons [52]. Moreover, in psychiatric disorders such as the post‐traumatic stress disorder, the LC signal was significantly elevated in NM‐MRI and related to the severity of clinical symptoms [53], highlighting the possibility that NM‐MRI could serve as a biomarker for catecholamine function [54].

Using NM‐MRI analysis, our study explored the function of LC in ET cognition. The involvement of the LC‐NE system in cognition has been extensively studied in several neurodegenerative illnesses like AD and PD. Previous investigations have shown that LC is an early site of tau deposition. Several autopsy studies have revealed that 50% of people in their 30s to 40s, when cortical disease is not visible, have hyperphosphorylated tau accumulated in their LC [55, 56, 57]. Unrelated to the number of neurons in the LC, a higher tangle burden in the LC is associated with a steeper cognitive deterioration [45]. To date, few studies have reported the role of LC‐NE system in ET [58]. A study showed a notable decrease in the urinary excretion and normal plasma levels of NE in ET patients (*n* = 40) [59], suggesting the possibility of noradrenergic dysfunction of patients of ET. However, one brain postmortem study with small samples found increased concentrations of NE in LC, nucleus dentatus, and cerebellar cortex of ET in comparison with normal controls while another one found similar concentrations of dopamine‐beta‐hydroxylase (a marker of noradrenergic neurons) in the LC of both groups [60, 61]. In addition to mainstream cerebellar pathology [62], some ET cases have been found brainstem Lewy bodies deposition, particularly in the LC [16, 63, 64]. Due to the small sample size and difficulty in replication, these results should be interpreted with caution. Propranolol, a non‐selective beta‐adrenergic receptor blocker, is the first‐line treatment in lessening the tremor symptom of most patients with ET [7]. Further study suggested the pharmacological effects may be associated with the blockage of peripheral beta‐2adrenergic receptors in the striate muscle [65]. Lv et al. found CNR_LC_ values decreased in patients of ET but no relationships between CNR_LC_ values and tremor severity [29], and our research also yielded similar results. Instead, we found that the ET‐MCI patients had the worse CNR_LC_ values, indicating that some pathological changes of LC contribute to the cognitive impairment and is independent of motor manifestations. The correlations between CNR_LC_ values with TMT‐A time and MoCA scores indicate the damaged integrity of LC would result in the complicated cognitive deficits. Regarding the negative relationship between cerebellar volumes and cognition, we anticipate that future studies with larger sample sizes will investigate this area further and consider the inclusion of the CCAS scale as part of a comprehensive cognitive assessment.

It is critical to recognize that the clinical phenotype of ET is diverse and that, in terms of differences in underlying causes or pathophysiology, ET‐plus may represent a state condition as opposed to a trait condition [8]. While the processes or pathways directly related to the disease may mediate cognitive impairments in ET, another possibility is that some ET cases may also exhibit a series of neurodegenerative diseases, such as AD, PD, or Parkinson plus disorders, which may lead to the cognitive impairment [66]. Besides, compared to early‐onset ET, late‐onset ET was more likely to develop cognitive decline, poor grip strength, and mortality [67, 68]. Therefore, “aging‐related tremor” seemed to be a more suitable term [69]. Undoubtedly, there is phenotypic heterogeneity within ET. In the future, it would be ideal to define subgroups based on their pharmacotherapeutic response profile, other biomarkers, or their genetic, physiological, or pathological bases [8].

## 5 Limitation

There are some limitations in our study. To be first, the involvement of LC in the spectrum of neurodegenerative disorder is a very interesting topic and many new technologies for measuring LC have emerged [70, 71, 72]. In our study, because of the slice thickness of 3 mm, manual segmentation is more stable than automatic registration, though it will result in less reproducible in the future. In addition, up to now there is no consistence among researchers whether the high signal intensity of LC was from neuromelanin or their high‐water content with magnetization transfer effect [73] and thus our findings should be interpreted with caution. It would be ideal to use NM‐MRI with high‐ or ultrahigh‐resolution or more updated approach (e.g., positron emission tomography, measures of noradrenergic transporter levels) to confirm our findings. Besides, as a cross‐sectional study with a relatively small sample, ours was unable to confirm NM changes in LC as a biomarker for the advancement of ET. To find out if CNR_LC_ could be used to predict ET's cognitive decline, more longitudinal studies with larger sample sizes and longer follow‐up periods are required. Finally, due to the relatively lower levels of formal education in our study, which is in line with other Chinese datasets [74], non‐Chinese cohorts with higher levels of formal education need to be cautious when applying our results.

## 6 Conclusion

In conclusion, we found that the integrity of LC was significantly decreased in ET patients and be more pronounced in ET‐MCI patients. Decreased integrity of LC was correlated with the severity of cognitive functions. We suggest that LC dysfunction may be a factor in attention and working memory function deficits as well as a more complicated form of cognitive decline in ET patients. These results provide further interpretations of the LC‐NE system in ET‐MCI patients and facilitate future work using more updated method of LC imaging to explore mechanisms underlying the cognitive decline. In addition, our study highlights the potential of LC imaging as a supplement to clinical data to support better diagnosis and early intervention in patients with cognitive impairment.

## Ethics Statement

This study has been approved by the Institutional Review Board and has therefore been approved performed in accordance with the ethical standards laid down in the 1964 Declaration of Helsinki and its later amendments.

## Consent

Informed consent was obtained from all individual participants included in the study.

## Conflicts of Interest

The authors of this manuscript declare no relationships with any companies, whose products or services may be related to the subject matter of the article.

## Supporting information


**Table S1.** The demographic, clinical, and CNR_LC_ characteristics of aMCI, naMCI.

## Data Availability

The data used in this study, obtained from The Second Affiliated Hospital, Zhejiang University School of Medicine, are available from the corresponding author upon reasonable request.
