# Supplementary material for: Locus Coeruleus Degeneration in Essential Tremor With Mild Cognitive Impairment: A Neuromelanin MRI Study
Source: CNS Neurosci Ther. 2025 Jan 8;31(1):e70214. doi: 10.1111/cns.70214 (PMC11707437; doi:10.1111/cns.70214)
Supplement: Supplementary file 1 — Table S1. The demographic, clinical, and CNRLC characteristics of aMCI, naMCI. [file CNS-31-e70214-s001.docx]

**Supplementary Table 1. The demographic, clinical, and CNR_LC_ characteristics of aMCI, naMCI.**

|  | aMCI (n = 20) | naMCI (n = 10) | *p* value |
| --- | --- | --- | --- |
| Age (years) | 60.49 ± 6.59 | 61.47 ± 6.98 | 0.709^a^ |
| Sex (M/F) | 11/9 | 5/5 | 0.796**^b^** |
| Education (years) | 8.45 ± 2.33 | 9.10 ± 2.56 | 0.491^a^ |
| CNR_LC_ | 2.17 ± 0.67 | 2.02 ± 0.59 | 0.549^a^ |

CNR_LC_: Contrast-to-noise ratio of the locus coeruleus; aMCI: amnestic MCI; naMCI: non-amnestic MCI; ^a^ Comparison performed using a parametric test; ^b^ Comparison performed using chi-square test.
